# Identifying Time Periods of Minimal Thermal Gradient for Temperature-Driven Structural Health Monitoring

**DOI:** 10.3390/s18030734

**Published:** 2018-03-01

**Authors:** John Reilly, Branko Glisic

**Affiliations:** Department of Civil and Environmental Engineering, Princeton University, Princeton, NJ 08540, USA; bglisic@princeton.edu

**Keywords:** Temperature-Driven Structural Health Monitoring (TD-SHM), thermal gradients, fiber optic sensors, prestressed concrete bridge

## Abstract

Temperature changes play a large role in the day to day structural behavior of structures, but a smaller direct role in most contemporary Structural Health Monitoring (SHM) analyses. Temperature-Driven SHM will consider temperature as the principal driving force in SHM, relating a measurable input temperature to measurable output generalized strain (strain, curvature, etc.) and generalized displacement (deflection, rotation, etc.) to create three-dimensional signatures descriptive of the structural behavior. Identifying time periods of minimal thermal gradient provides the foundation for the formulation of the temperature–deformation–displacement model. Thermal gradients in a structure can cause curvature in multiple directions, as well as non-linear strain and stress distributions within the cross-sections, which significantly complicates data analysis and interpretation, distorts the signatures, and may lead to unreliable conclusions regarding structural behavior and condition. These adverse effects can be minimized if the signatures are evaluated at times when thermal gradients in the structure are minimal. This paper proposes two classes of methods based on the following two metrics: (i) the range of raw temperatures on the structure, and (ii) the distribution of the local thermal gradients, for identifying time periods of minimal thermal gradient on a structure with the ability to vary the tolerance of acceptable thermal gradients. The methods are tested and validated with data collected from the Streicker Bridge on campus at Princeton University.

## 1. Introduction

Temperature poses a challenge in Structural Health Monitoring (SHM). Temperature effects in a healthy structure can affect the monitoring features (strain, modal parameters, etc.) as much as or more than unusual structural behavior [1,2,3]. Current design codes for bridges have considerations for temperature changes and both linear and non-linear thermal gradients [4,5] but these specifications are not exhaustive for thermal behavior in bridges. For example, the sun can heat up parts of a bridge to much more than ambient temperatures, an event usually ignored in design codes [6,7], and the thermal response of structures can be highly variable, depending on climate, structural system, and other factors [8]. Many areas of SHM research filter out temperature effects, as they can affect the principal parameters being monitored, such as mechanical strain or modal parameters. For example, Laory et al. examined the effects of filtering out seasonal variations of temperature on damage detectability in a numerical bridge model [9], while Worden et al. employed a variety of sophisticated analytic techniques, including robust regression, cointegration, and the Mahalanobis distance squared to remove environmental influences from modal parameters in free vibration analysis [10,11,12,13]. Current SHM treats thermal influences as an unavoidable environmental side effect that is to be filtered out. However, the effects of temperature, while sometimes necessary to filter out to isolate other parameters, may contain important information and reveal critical aspects of structural behavior [14].

Contrary to traditional SHM techniques, Temperature-Driven Structural Health Monitoring (TD-SHM) focuses on temperature as the principal driving force in structural behavior and analysis, treating generalized temperature changes as an input affecting a generalized strain and generalized displacement output. The term “generalized temperature” encompasses temperature change at a point, and any algebraic combination of temperature changes at different points (e.g., linear thermal gradient in a cross-section). Similarly, the term “generalized strain” encompasses strain value at a point, and any algebraic combination of strain values at different points (e.g., curvature in cross-section). Finally, “generalized displacement” encompasses displacement at a point, rotation at a point, and algebraic combinations of these parameters. To simplify presentation in further text, the word “generalized” will be omitted and used only when needed to clarify the subject. These three measurable quantities—temperature, deformation, and displacement—will form 3D signatures for different parts of the structure, with the expectation of sensitivity to changes in structural behavior [1,14]. An important challenge in TD-SHM is the fact that temperature change in one part of the structure can generate mechanical strain and displacement in another part of the structure, and thus this latter can falsely be interpreted as unusual structural behavior (e.g., damage). Numerical modelling or system identification based on all possible thermal inputs is impractical, as those should include the thermo-mechanical properties of the structure (which are difficult to assess on-site), the large scale of structures and their geometrical complexity, as well as site-specific environmental effects such as partial shadow due to trees, variable proximity to land or water, etc. By deriving and analyzing the expressions of structural behavior under thermal actions, it is proven in this paper that an appropriate approach could be to use only the data sets collected during the times when thermal gradients in the structure were minimal. Thus, this work seeks to address the above challenge by identifying time points when the thermal gradients on a monitored structure are minimal, and use data collected at these time points to analyze structural behavior. Two simple yet practical classes of methods are proposed, showing that they identify significantly different sets of time points, with different values characterizing parameters describing structural behavior (i.e., 3D signatures). This is an important insight that demonstrates the complexity of the presented problem and calls for future research. To achieve TD-SHM, first the influence of thermal gradients has to be minimized.

Contemporary research on thermal gradient influences on structures has produced a variety of results. Few works are cited here to emphasize the challenge of interpretation of data collected while thermal gradients were present on the structure. Hedegaard et al. created a two-dimension finite element mesh and were able to tailor the model to predict structural response to thermal gradients based on temperature and strain readings from the I-35W St. Anthony Falls Bridge [8]. Despite this ability to create an accurate model, Hedegaard and other researchers noted that the thermal response of a structure is highly variable for each bridge, dependent on climate, location, and the properties of the structure [8,15]. Non-linear thermal gradients can induce stress in even cases of free expansion or rotation, contrary to normal assumptions [16]. These non-linear thermal gradients can be difficult to clearly relate to structural response. For example, Kromanis employed a data-driven strategy (as opposed to model-based) on distributed temperature measurements to accurately predict the response of the structure after a set amount of training data [17].

For the purposes of this paper, thermal effects to the beam-like structure, its parts, or its cross-sections could be defined in terms of global thermal change and local thermal gradients (see details in the next section). Thermal gradients in a structure are time-dependent, occurring while heat enters or leaves the material. Due to the non-instantaneous nature of heat transfer, depending on the thermal inertia of the material, thermal gradients are frequently non-linear. These non-linear gradients can cause non-linear thermal strain distributions, which can result in bending and non-linear stress and strain distribution within the cross-sections, obscuring the effects of thermal change and making data analysis complex and in many cases even inconclusive [16,18,19]. Thus, identifying time periods of minimal thermal gradients will allow for the establishment of a relationship (signature) between the temperature change and resulting strains and displacements, and enable direct comparison between the relationships occurring at different times. Hence, this paper focuses on an initial component of TD-SHM: the creation of methods for the identification of time periods of minimal thermal gradients in a structure.

The main challenge in the creation of these methods is that due to the large scale of many structures and the varying exposure to temperature, real structures are practically never in a state of zero thermal gradients. Thus, a certain level of thermal gradients has to be tolerated, which raises a trade-off challenge. If a large range of thermal gradients is tolerated, a substantial amount of data will fall into that range, but the uncertainty in data analysis will be significant. On the other hand, if a small range of thermal gradients is tolerated, the data that falls into that range might be very small and insufficient for effective data analysis. While several methods were considered in the course of this research, such as the standard deviation of temperatures and median local gradient [20], the two approaches that were identified as the most promising ones are presented in this paper: evaluating the range of temperature measurements on the structure and the distribution of local gradients on the structure. Their performance is evaluated using data from a real structure, Streicker Bridge, on the Princeton University campus.

## 2. Temperature Distribution and TD-SHM

For any temperature distribution within a beam-like structure under linear-theory assumptions, there is a unique deformation and displacement distribution. Thus, theoretically, if a very dense network of temperature, strain, and displacement sensors were applied to a structure, it would be possible to accurately determine the relationship between (generalized) temperature, strain, and displacement, and create their 3D diagrams, i.e., TD-SHM signatures.

**Figure 1:**
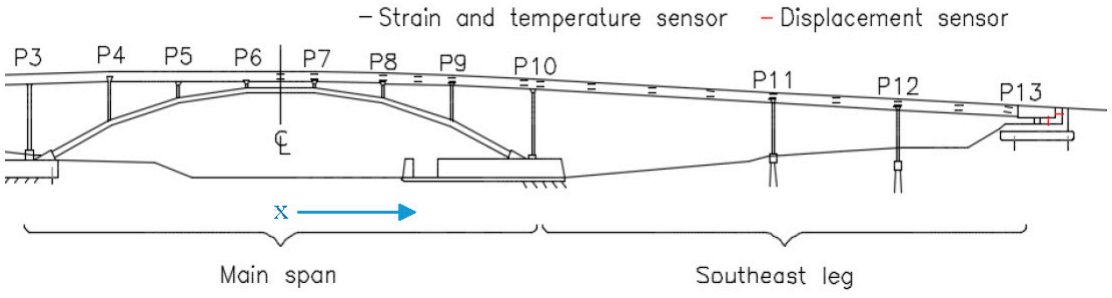
Example of a beam-like bridge instrumented with a chain of parallel sensors along the deck and displacement sensors at extremity (Streicker Bridge).

However, in practical applications the available sensor networks might not be sufficiently dense. Frequently, few sensors are installed within the cross-section of a beam member, and only a few cross-sections are instrumented along the beam member. A typical example is given in Figure 1, where a chain of parallel strain and temperature sensors is installed on the structure, and displacement sensors are installed only at the expansion bearing at Pier 13 (P13 denotes the bridge pier or supports).

In Figure 1, each instrumented cross-section contains two sensors, one at the top and the other at the bottom of the cross-section, with approximately five meters between instrumented cross-sections. This limitation in sensor coverage results in increased uncertainty in determining TD-SHM signatures. This research aims to reduce the uncertainty by creating methods to conveniently choose data to be used in analysis; namely, choosing time periods of minimal thermal gradient on the structure in order to increase the linearity in the temperature–strain–displacement relationship and thus reduce the uncertainty in the TD-SHM signatures. In the following text, planar structures are considered for simplicity of presentation. The planar case can be expanded to the spatial case by adjusting equations to account for bending moments and thermal gradients in the horizontal transversal direction.

**Figure 2:**
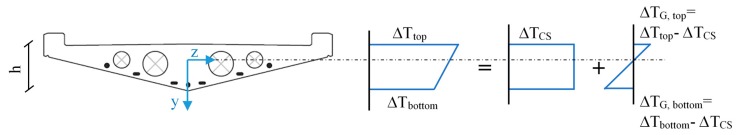
Example of a bridge cross-section with linear temperature distribution.

Thermal gradients in a beam-like structure induce curvatures in cross-sections and non-uniform elongation along the length and may or may not generate stresses depending on structural system and boundary conditions. The largest effects from these thermal gradients appear from vertical thermal gradients acting across the depth of the cross-section of a beam. In the case of steady temperature conditions, the thermal gradients are linear. Linear temperature distributions in a cross-section can be decomposed into temperature change and vertical thermal gradient, as shown in Figure 2.

The linear temperature distribution shown in Figure 2 deconstructs into the combination of a uniform temperature change Δ*T_CS_*, corresponding to temperature change at the centroid of stiffness of the cross-section, and a linear thermal gradient *G*_Δ*T*_ = *(*Δ*T_bottom_* − Δ*T_top_)/h*, which is equal to the difference between temperature variations at the bottom and the top of the cross-section divided by the depth of the cross-section *h*. Linear temperature distributions induce elongation along the beam and curvature in cross-sections, but not deplanations of the cross-section. Assuming that a linear temperature distribution Δ*T*(*x*,*y*) is present on a beam-like structure, the change in strain Δ*ε_total_*(*x,y*) at a point with coordinates *x* along the beam and *y* from the centroid of the section, due to the linear temperature distribution, can be expressed as follows for a plane-stress case:

(1) $$\Delta \varepsilon_{total}\left(x,y\right)=\Delta \varepsilon_{mech.}\left(x,y\right)+\Delta \varepsilon_{T}\left(x,y\right)=\frac{N_{\Delta T}\left(x\right)}{EA}+\frac{M_{\Delta T}\left(x\right)}{EA}y+\alpha_{T}\Delta T_{CS}\left(x\right)+\alpha_{T}G_{\Delta T}\left(x\right)y$$

where Δ*ε_mech._*(*x*,*y*), *N*_Δ*T*_, and *M*_Δ*T*_ represent thermally generated mechanical strain, normal force, and bending moment, respectively; *α_T_* and *E* represent the thermal expansion coefficient and Young’s modulus of the material; *A* and *I* represent area and moment of inertia of the cross-section; and Δ*T_CS_* and *G*_Δ*T*_ represent temperature change at the centroid of stiffness and thermal gradient in the cross-section with coordinate *x*.

Note that *N*_Δ*T*_ and *M*_Δ*T*_ in a cross-section depend not only on Δ*T_CS_* and *G*_Δ*T*_ at that cross-section, but also on Δ*T_CS_* and *G*_Δ*T*_ at other cross-sections, and on the structural system and boundary conditions. Thus, the relationship between temperature and strain at a point is not linear in the general case. However, if no longitudinal and transversal (vertical) thermal gradients are present, i.e., Δ*T_CS_*(*x*) = Δ*T* = *constant* and *G*_Δ*T*_(*x*) = 0, then the relationship between strain *Δε_total_*(*x*,*y*) and uniform temperature change in the structure Δ*T* becomes linear. In that case, the coefficient of linearity depends on the thermal expansion coefficient *α_T_*, the Young’s modulus *E*, the structural system (boundary conditions), and the geometrical properties of the structure. Similar statements can be derived for any generalized deformation parameter (e.g., curvature).

The change in generalized displacement Δ*δ*(*x_δ_*) at a point with coordinate *x_δ_* along the structure can be determined using the principle of virtual work, by applying the corresponding generalized unit force, determining the virtual normal force and virtual bending moment distributions, and applying the formula (influence of shear force is neglected):

(2) $$\Delta \delta \left(x_{\delta }\right)=\underset{l}{\int }\frac{N^{v}\left(x\right)N_{\Delta T}\left(x\right)}{EA\left(x\right)}dx+\underset{l}{\int }\frac{M^{v}\left(x\right)M_{\Delta T}\left(x\right)}{EI\left(x\right)}dx+\underset{l}{\int }N^{v}\left(x\right)\alpha_{T}\Delta T_{CS}\left(x\right)dx+\underset{l}{\int }M^{v}\left(x\right)\alpha_{T}\Delta G_{\Delta T}\left(x\right)dx$$

where the superscript *v* indicates influences due to virtual unit generalized force, and *l* indicates integration over all beams in the structure.

Note that in structures consisting of several beams, *N^v^* is constant along each beam, and *M^v^* is linear or bi-linear along each beam. Analysis similar to the strain–temperature relationship demonstrates that in the absence of gradients the relationship between generalized displacement Δ*δ*(*x_δ_*) and uniform temperature change in the structure Δ*T_CS_* becomes linear. Based on the above considerations, the relationship between uniform temperature change, generalized strain, and generalized displacement is linear, and the graph of this relationship, i.e., its TD-SHM signature, is a segment of a line or planar figure (i.e., 3D linear).

**Figure 3:**
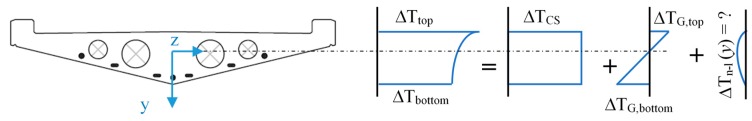
Example of a bridge cross-section with non-linear temperature distribution (causing non-linear thermal gradient).

In reality, temperature distributions are non-linear (causing non-linear gradients) because of the non-instantaneous nature of the heat transfer through the material of the section, which is a consequence of thermal inertia and low diffusivity, particularly in the case of concrete. In addition to the global non-linearity of TD-SHM signatures introduced by linear thermal gradients (see Equation (2)), non-linear thermal gradients cause non-linear strain distributions within the cross-section and additional internal stresses [21]. This is schematically shown in Figure 3.

**Figure 4:**
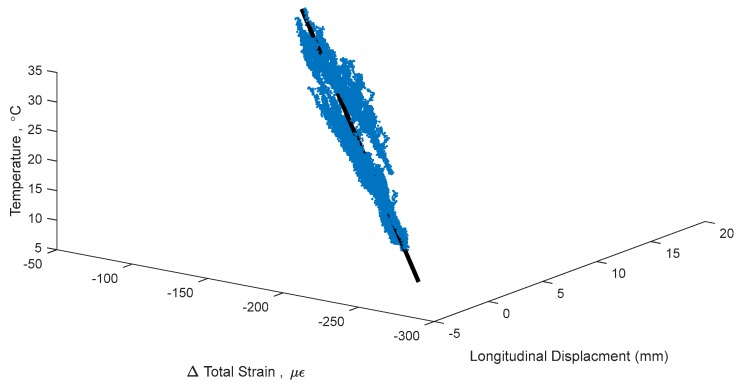
Example of a 3D signature of temperature, strain, and longitudinal displacement with best fit line, taking into account data resulting from non-linear temperature distributions.

This non-linear thermal gradient causes an error when creating TD-SHM signatures. The error is directly correlated with the amplitude of non-linearity Δ*T_n−l_*(*y*) (see Figure 3), which is in turn correlated to the difference in temperature between the top and bottom of the cross-section and to whether the bridge is cooling or heating [4,5]. An example TD-SHM signature created for Streicker Bridge (see Figure 1) using a full set of data collected in spring 2016, i.e., containing some data resulting from non-linear temperature distribution, is given in Figure 4. Figure 4 shows a signature using strain and temperature at the centroid of the section located at Pier 12, and displacement readings taken at the abutment of the South-East leg (see Figure 1). This signature is used as an example in this text to validate the methods of identifying time periods of minimal thermal gradients. Final formulation of TD-SHM will evaluate 3D signatures at multiple locations in the structure. The signature is shown from different angles to emphasize its non-linear shape and noise related to non-linear temperature gradients. 

**Figure 5:**
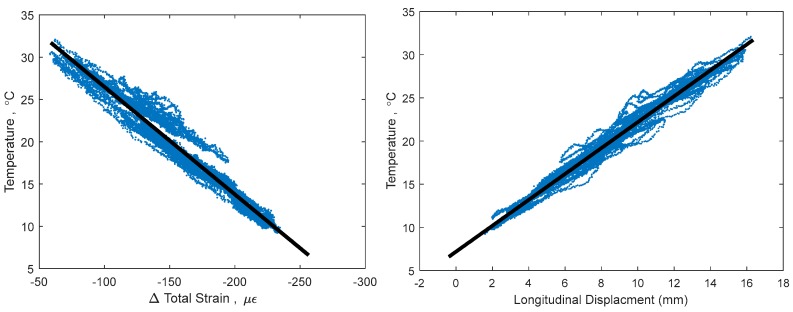
2D projections of 3D signature, keeping temperature as the vertical axis.

Figure 4 shows the 3D signature of temperature, strain, and longitudinal displacement, along with best fit line, and Figure 5 shows two projections of the signature to help present the three-dimensional shape. While there is a definite trend and linearity to the signature, it is obscured by noise due to large, non-linear thermal gradients in the structure. There is also some bi-linear behavior, seen in Figure 5 in the temperature and strain plot. This bi-linearity could be caused by thermal gradients in the structure, or by some other, currently unknown effect. The best fit line provides a method to compare the effectiveness of the different minimum gradient filters, by examining the vector (**n**) and intersection point (A) of the line, the coefficient of determination (*R*^2^), and the standard error (σ) of the best fit line. The vector of the best fit line and intersection point provide a description of the linear relationship between temperature, strain, and displacement, and are also sensitive changes in structural behavior. Higher *R*^2^ and lower σ would show a more accurate estimation of this behavior, increasing the expected ability to determine smaller changes in this structural behavior [1,14]. The vector of the best fit line of the entire data set (including non-linear thermal gradients) in Figure 4, is **n** = [0.084, 0.990, 0.126], relating to [displacement, strain, temperature]. This vector describes the linear relationship between the three parameters: for every 0.126 °C change in temperature, there is a corresponding 0.99 µε change in strain and 0.084 mm change in displacement. Boundary conditions geometry of the structure, and material restraints all have a strong influence on these 3D signatures; the linear relationship identified captures these effects in the vector of the line. Detailed analysis of each of these influences is out of the scope of this paper but will be performed in the next stage of the project when TD-SHM would be fully formulated and tested. The *R*^2^ value of the best fit line in Figure 4 is 0.989, and the standard error is 1.351. To simplify presentation, the intersection point (A) of the best fit line is not analyzed (the focus of this paper is minimization of thermal gradients, and thus full development of TD-SHM is out of the scope of the paper and will be addressed in the future work).

A change in structural behavior is expected to result in a change in the vector **n** (and intersection point A); however, this change can be unnoticed if the standard error is big or vector n is not well defined. For example, it is unclear whether the bi-linear signature in Figure 4 and Figure 5 indicates damage, or is simply the result of non-linear thermal gradients, and thus should be discarded. Identifying time periods of minimal thermal gradient in the structure is thus expected to provide the data that shapes TD-SHM signatures into linear or planar figures in 3D space, and at the same time minimizes the possible error (from non-linear thermal gradients) in identification of unusual structural behaviors.

## 3. Methods for Determination of Minimal Thermal Gradients

**Figure 6:**
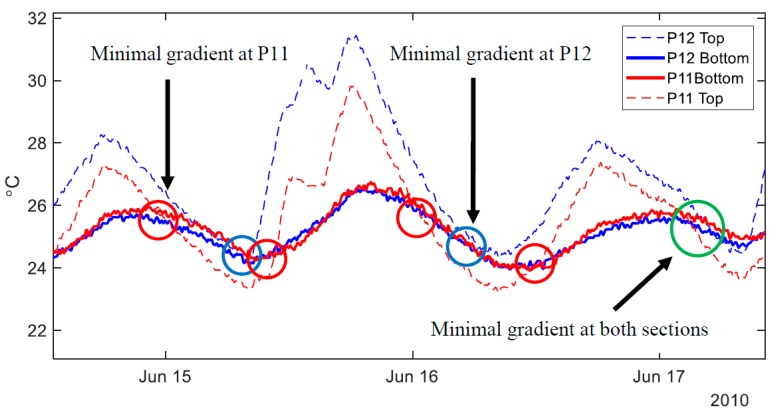
Example of temperature variation over two days in two cross-sections of Streicker Bridge (top and bottom sensors in cross-sections at P11 and P12).

Using the data when an approximately uniform temperature is present across the structure will minimize the influence of non-linear thermal gradients, allowing for planar TD-SHM signatures. However, moments in time when the temperature is approximately uniform across the bridge are scarce. As an illustration, Figure 6 shows temperature fluctuations at the top and bottom of two cross-sections of the Streicker Bridge (see Figure 1) over two days.

Figure 6 shows the temperatures recording at the top and bottom of two cross-sections (P11 and P12, see Figure 1) on the Streicker Bridge over two days. While each cross section individually experiences uniform temperatures at two instances per day (red circle corresponds to times with minimal thermal gradient at P11, and blue for P12), Figure 6 shows that there is no moment in time over the given two days when the temperature was perfectly uniform at both observed cross-sections. The sections come closest to achieving zero thermal gradient at the end of the second day (green circle) but still do not reach a uniform temperature. This difficulty in achieving zero thermal gradient over the two cross-sections in the bridge highlights the improbability of the entire bridge having a uniform temperature. These moments in time are extremely scarce and significantly reduce the data available for creating TD-SHM signatures. Because of the unlikelihood of finding an absolutely uniform temperature across an entire bridge, some level of non-uniformity must be tolerated. Higher levels of non-uniformity imply more data available for analysis, but also more error in the analysis, and thus an acceptable balance between these two opposite objectives must be found. In this paper, two classes of methods are proposed to identify time periods of minimal thermal gradient: one using the range of temperature measurements on the structure, and the other using the distribution of local thermal gradients. The methods are presented in detail in the next subsections. Their advantages and limitations are discussed.

### 3.1. Maximum Range

The Maximum Range (MR) methods stipulates that the total range of temperatures on the structure at an observed moment in time needs to be below some chosen value for the MR, as shown in Equation (3). *T_Max_* and *T_Min_* represent the maximum and minimum temperature on the structure, all sensor measurements accounted.

(3) $$T_{Max}-T_{Min}<\mathrm{MR}$$

Choosing the value of the MR needs to balance the amount of time points identified (larger MR needed) while still minimizing the tolerance of thermal gradients (lower MR needed).

An important challenge of the MR method is its susceptibility to a single or few outlying temperature measurements. If the entire structure rests at a uniform temperature (within MR) with the exception of one or few locations outside the MR, then the structure would not be identified as having a minimal thermal gradient. However, given that the remainder of the structure is within the MR, these outlying temperatures cannot be excessively far from MR under usual daily temperature variations (for example, if most of the temperatures are within MR = 5 °C, then it is not expected for outlying temperature at one point to be out of range for much larger value, e.g., 10 °C), and thus including them in the analysis would not result in an excessive error. In addition, based on Equations (1) and (2), if one or few locations have temperatures different from the remainder of the bridge, this difference will influence local behavior around these points rather than global behavior, which in turn will result in a small error in distant TD-SHM signatures. Hence, to address the challenge of small data sets with truly uniform temperature, a modified MR method is created where there is a tolerance of certain percentage (e.g., 5%) of outliers outside of the MR. The acceptable value of MR and the percentage of acceptable outliers depend on the structural system, the scale of the structure, and the number of sensors installed on the structure, and can be determined based on a sensitivity study, as shown in the next section with results.

### 3.2. Local Gradient

The local gradient (LG) methods examines the local thermal gradients (thermal gradients through the cross-section) at several cross-sections. For this application (Streicker Bridge with horizontal deck), vertical thermal gradients through the cross sections are considered as they have the largest effect on the structure, though other structures may have different dominant directions of gradient in the section. The local vertical thermal gradient in a cross section (*G*Δ*_T_*(*x*)) is defined in Equation (4), where *h* is the distance between the measurements Δ*T_bot_* and Δ*T_top_*.

(4) $$\frac{\Delta T_{bot}-\Delta T_{top}}{h}=G_{\Delta T}\left(x\right)$$

Two methods were identified using this metric. First the Maximum Local Gradient (MxLG) method identifies time periods where every cross-section on the structure has an absolute local gradient under some chosen MxLG bound. If the absolute value of every local thermal gradient on the structure is less than the chosen MxLG, then the temperature state of the structure is deemed suitable for TD-SHM method. This method is fundamentally different from MR. Beyond using gradients rather than raw temperatures, the MxLG method only compares each location on the bridge to its corresponding sensor at its cross section only (e.g., bottom sensor with top sensor) and not with every other sensor. Thus, while temperature gradient is minimized across the cross-section, there could be some temperature change along the centroid line of the structure, i.e., Δ*T_CS_*(*x*) ≠ *constant*.

Next, the Mean Local Gradient (MeLG) method identifies time periods where the mean absolute value of all local thermal gradients on the structure is under some chosen bound. A mean local gradient closer to zero directly corresponds to the minimization of all cross sectional thermal gradients on the structure. This method is affected by each local gradient on the structure, while MxLG is only concerned by the individual maximum local gradients on the structure. 

Minimization of local gradients, i.e., G_Δ*T*_(*x*) ≈ 0, removes the last terms from Equations (1) and (2). In addition, it minimizes the values of *N*_Δ*T*_ and *M*_Δ*T*_ since they are result of only axial thermal elongations of the beams (no thermal curvature due to gradients). Thermal elongations are in turn a consequence of temperature change along the beam, and non-linear effects will arise only if the change is abrupt at some points, which is not expected to happen (similar comment applies as in case of MR). Thus, the TD-SHM signature is expected to be only slightly “distorted” from the linearity. Both MxLG and MeLG parameters can be evaluated based on sensitivity study, as shown in the next section.

### 3.3. Process for Identifying Time Periods of Minimal Thermal Gradient

**Figure 7:**
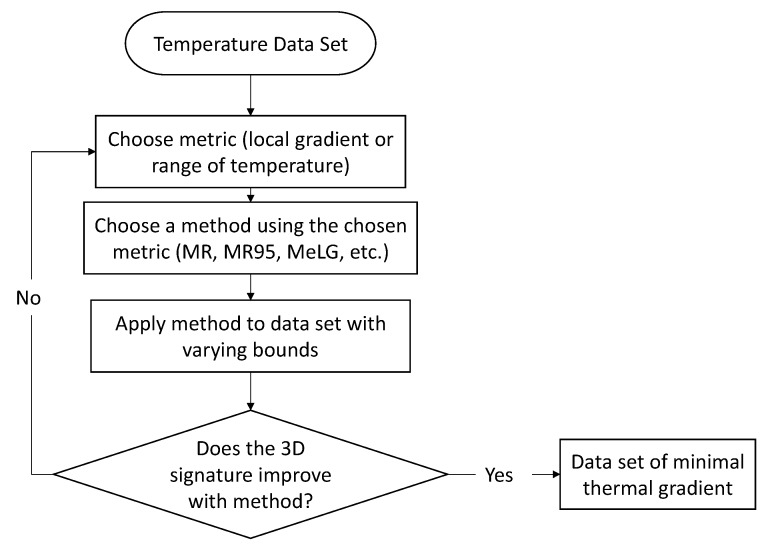
Flowchart describing process of using methods for identifying time periods of minimal thermal gradient.

The entire process of identifying time periods of minimal thermal gradient is shown in Figure 7.

Starting with the total temperature data set, the metric and method for identifying minimal thermal gradients needs to be chosen. This choice can depend on the individual monitoring system and the monitored structure. Next, the chosen method is applied to the data set using a variety of bounds. It can be difficult to predict the bounds that will maintain the minimization of thermal gradients, while producing a large enough basis of time points. To choose a bound and validate the method, the 3D signature of the total data set is compared to the 3D signature of the time points of minimal thermal gradient. If there is an improvement in the linearity (coefficient of determination and standard error) of the 3D signature, the method is deemed valid. The final bound is chosen based on the stabilization of the vector of the best fit line or plane, as discussed later in Section 4. If the 3D signature does not improve with the application of the chosen method, a different metric or method can be chosen to repeat the process. 

## 4. Evaluation of Methods

Data collected from the Streicker Bridge on campus at Princeton University is used for exploration and assessment of the two classes of methods proposed in previous section. The Streicker Bridge is a complex structure consisting of a deck-stiffened arch and four approaching continuous beam girders (called legs). The bridge was built in 2009 and equipped with over 100 fiber optic temperature and strain sensors embedded in the concrete deck of the main span (deck-stiffened arch) and one of the legs (South-East leg). A fiber optic system was chosen for its insensitivity to electromagnetic fields, reliability in demanding environments, and their potential for both distributed and discrete sensing. A simplified monitoring plan is shown in Figure 1, with a focus on the Fiber Bragg Grating-based sensors, relevant in this work. Each instrument is comprised of two individual sensors: one strain-insensitive temperature sensor, and one temperature-compensated strain sensor. These sensors are arranged in a parallel topology at critical cross sections: column beam intersections and mid spans. This topology with one sensor at the top and one at the bottom of the sections captures the largest change in strain in the sections, while providing information on the thermal gradients and curvature in the section. Having more sensors across the cross-section would be more beneficial for full understanding of thermal behavior; however, economic limitations imposed the installation of only two sensors per cross-section. Additional displacement sensors were installed in 2016 at the abutment of the South-East leg. The instrumented length of the bridge is approximately 58 m and uncertainty in temperature monitoring is estimated to be ±0.2 °C [22]. Sensor drift is a concern with all long-term monitoring projects and could significantly affect the results of this work. Sensor drift due to manufacturing error has been previously identified and addressed in earlier work on the Streicker Bridge [23]. Measurements were taken statically every five minutes. Temperature changes are influenced by a variety of environmental factors: ambient weather and exposure to wind, rain, snow, and sun. Strain changes are primarily influenced by temperature (through thermal expansion and thermally generated mechanical strain). The structure was built in 2009 and is beyond the majority of rheological strains (creep and shrinkage are mostly stabilized). The influence of transient mechanical loads is minimized by recording strain as the average value of ten measurements taken every five minutes [19]. Each measurement session included all sensors. More detailed information on the Streicker Bridge and monitoring system can be found in [24].

Both presented classes of methods MR and LG demonstrated the capability of identifying time periods of minimal thermal gradients on the Streicker Bridge, but they are different in their results, as shown in the following subsections.

### 4.1. Evaluation of Maximum Range Method

**Figure 8:**
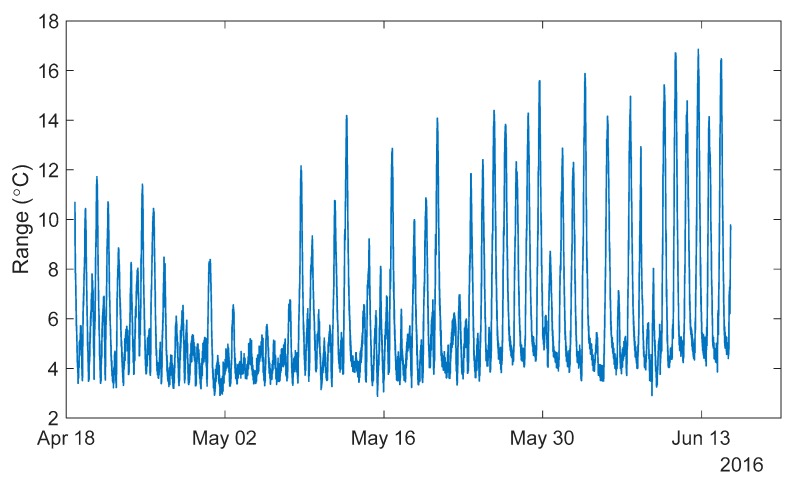
Range of temperature measurements from spring 2016.

Figure 8 shows the temperature ranges recorded from 18 April to 15 June 2016 on the Streicker Bridge with temperature sensors in the South-East leg.

Each point in Figure 8 represents the range of temperatures within each time point of measurement taken over twenty sensors in the concrete deck of the South-East leg of Streicker Bridge. A lower range in temperature indicates a more uniform temperature across the bridge. The minimum range shown in the figure is approximately 2.9 °C and the maximum is 16.9 °C. While the lowest ranges were under 3.0 °C, taking this value as the MR bound would not be useful—a very small number of time points would be found (see Table 1). Too small a number of points will skew the 3D signature, allowing noise or random effects to dominate, rather than the thermal behavior of the structure, and preventing any definitive estimation for the vector of the best fit line. Thus, a parametric study was made to establish the MR value which would be optimal, i.e., as small as possible to identify state of the bridge with temperature as close to uniform as possible, yet providing sufficiently large datasets for TD-SHM analysis. Table 1 summarizes the number of time points identified based on the given range of temperature measurements on the structure during spring 2016 (see Figure 8).

**Figure 9:**
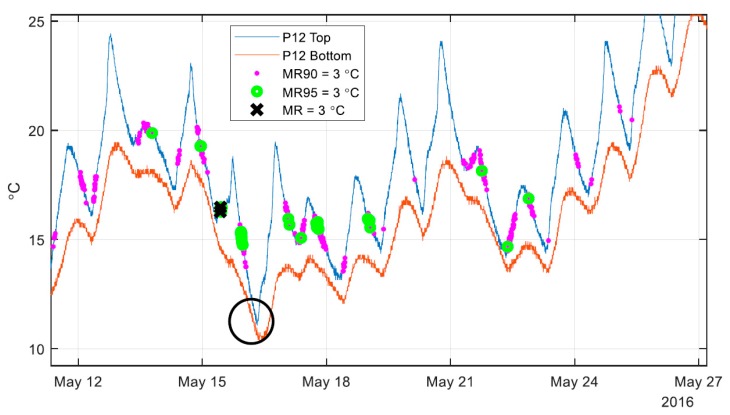
Points considered as having minimal gradients, observed using MR methods.

The spring 2016 data set contains 16,641 total time points of temperature distributions. MR95 seeks to retain 95% of the sensors within the given range, while MR90 retains 90%. The amount of outliers to allow was chosen based on the number of sensors available (95% excludes one out of 20 sensors, while 90% excludes two out of 20 sensors in Streicker Bridge). Table 1 shows that in many cases the range of temperatures is dominated by two or fewer outliers (10% of sensors or less), as allowing these outliers greatly increases the number of time points for each bound. An interesting pattern arising from the data set shown in Table 1 is that an increase of MR for 0.5 °C adds a similar number of time points as adding a tolerance of 5% of outliers. For example, starting at a MR of 3.5 °C (438 time points) and either increasing the range by 0.5°C (MR = 4 °C, 2748 time points) or adding a 5% tolerance of outliers (MR95 = 3.5 °C, 2773 time points) results in a similar number of time points of minimal thermal gradient. To illustrate the effects of increasing the tolerance of outliers, Figure 9 shows the three levels of outlier tolerance for two weeks in the spring of 2016.

**Figure 10:**
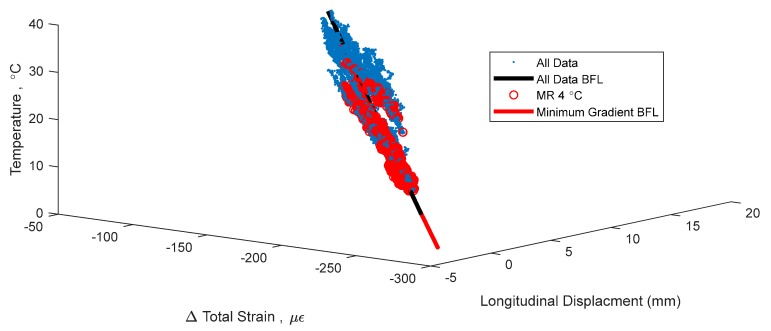
3D signature example with best fit line (BFL) for time points with minimal thermal gradient, identified using MR method.

Figure 9 shows the temperature sensors at pier 12 at the top and bottom of the cross section as a reference while highlighting the time points identified as having minimal thermal gradient. Only two weeks of data are shown, in order to illustrate the effect of allowing outliers with the MR method, while Table 1 refers to the entire data set stretching from 18 April to 15 June 2016. Each point with a black “X” shows a time point where the range of all sensors (not just the two sensors shown above) is below 3 °C. The MR method prioritizes the entire structure over any individual section, as can be seen above in at 16 May in the black circle. There is a time period with very small thermal gradients at pier 12, but that time period has no time points identified as having minimal thermal gradient, due to a more global non-minimized state of the thermal gradients on the structure. Allowing 5% outliers (MR95) will keep all of the time points without outliers (MR) but identify a larger set of time points with minimal thermal gradient. This tradeoff between identifying a larger set of time points with minimal gradients and compromising the minimization of the thermal gradients can be seen in the best fit line of the 3D signature for the structure shown in Figure 10.

**Figure 11:**
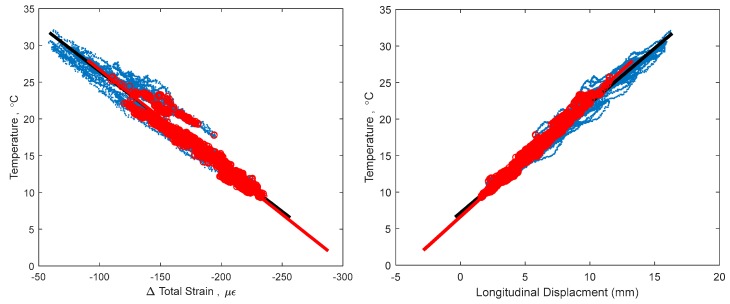
2D projections of 3D signature, keeping temperature as the vertical axis. Red indicates time points of minimal thermal gradient found using MR = 4 °C/m, and red line indicates best fit line for minimal gradient data set, as in Figure 10.

Figure 10 shows the same 3D signature in Figure 4, but with time points of minimal thermal gradient in red. For clarity, Figure 11 shows two 2D representations of the same 3D signature as in Figure 10. Only one method is shown in Figure 10, but Table 2 summarizes some statistics of the best fit lines for the different methods, including the vector of the best fit line (**n**), the coefficient of determination (*R*^2^), and the standard error (σ). The vector of the line describes the relationship between temperature, strain, and displacement, and this vector is expected to behave as a damage sensitive feature (in addition to the coefficient of determination and the standard error). Thus, an accurate evaluation of the vector **n** would enable sensitive detection of unusual behaviors. Results in Table 2 lead to several conclusions:

1. Each metric above shows a better linear fit in terms of coefficient of determination and standard error than the total data set (without minimum gradient filters), showing an improvement in the accuracy of the best fit line.
2. The first component of the vector **n** is significantly different for MR with bound 3.0 °C (0.097) from any other MR bound shown in Table 2; this shows that there is an insufficient number of points used in calculating the vector **n** (only 15 points).
3. For all other methods, the first component of the vector is a stable value at 0.080 ± 0.001 using the minimal gradient filters, the second component is very stable at 0.989, and the third component has some variation around 0.127 ± 0.003. These values for the vector of the best fit line fluctuate when there is a low number of time points forming the signature but stabilize as the number of time points identified increases. This consistency in the vector can be seen in MR with bounds 4.0 °C and 4.5 °C where the values are the same for the second and third component and 1% different in the first component, making confidence that any significant variation in any component of vector **n** from these values can be interpreted as unusual structural behavior in TD-SHM.
4. The stabilized vector of the best fit lines for minimal thermal gradient discussed in Point 3 (0.080, 0.989, 0.127) shows a more restrained section than with the vector of the total data set (0.084, 0.990, 0.126). For a larger change in temperature (0.127 °C), there are smaller resulting changes in strain (0.989 µε) and displacement (0.080 mm). This systematic difference, especially in the first component, of the vector of the best fit line shows that filtering out thermal gradients from the structure identifies a difference in the temperature–strain–displacement relationship.

The MR 3.0 °C bound only identified 15 time points of minimal thermal gradient; so few points compared to the other metrics results in a less reliable best fit line. Allowing some outliers (MR95 and MR90) greatly increases the number of time points identified but does not significantly reduce the quality of the best fit line. The standard error increases with the allowance of some outliers, but the *R*^2^ increases slightly. At the 3.5 °C bound, again the fit does not deteriorate with the allowance of outliers when comparing MR to MR95. This provides validation for the allowance of some small amount of outliers when identifying time periods of minimal thermal gradient. The exact number of points needed to provide an accurate description of the temperature–strain–displacement relationship will depend on the structure and specific application of the minimal gradient filters but can be identified by employing a range of methods (as above) and looking for a stabilization of the vector of the best fit line, while keeping standard error low and coefficient of coefficient of determination high.

### 4.2. Evaluation of Local Gradient Methods

**Figure 12:**
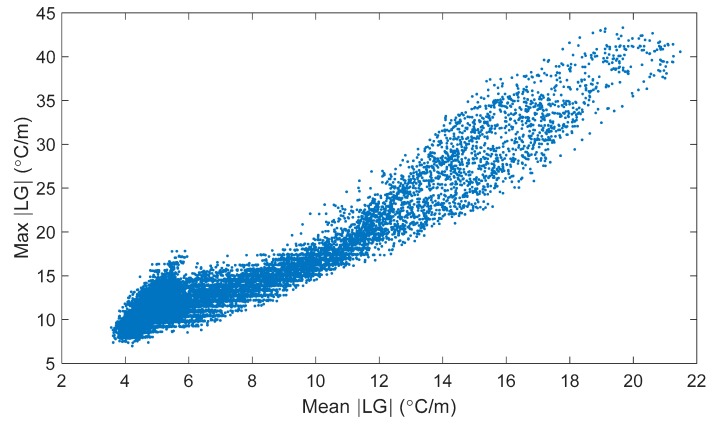
Maximum vs. mean absolute local thermal gradient.

Figure 12 shows the maximum vs. mean absolute local gradient found at each time step of recorded data in the spring of 2016. Each point in Figure 12 represents a single time point during the spring of 2016. The two methods identify different sets of time points—time points with a maximum gradient on the bridge under 10 °C/m could have a mean gradient from near 3.5 °C/m to more than 6 °C/m. To illustrate the magnitude of the thermal gradients in Streicker Bridge, a difference between the bottom and top sensors of −5 °C (if top is hotter, difference is negative) divided by a distance between sensors of approximately 35 cm would give a local thermal gradient of approximately 14 °C/m. Figure 12 shows that there is frequently at least this level of gradient on the bridge.

Table 3 summarizes the number of time points identified for each level of MxLG and MeLG.

**Figure 13:**
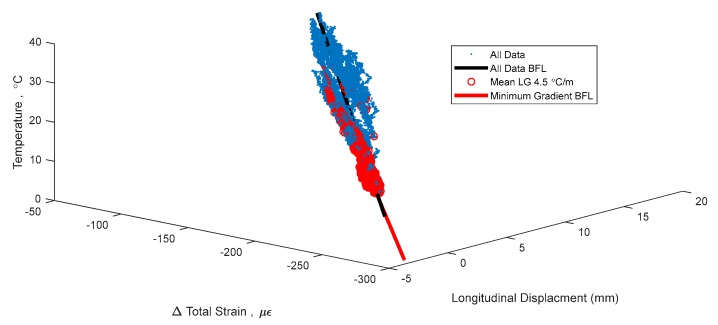
3D signature with best fit line (BFL) for time points with minimal thermal gradient, identified using (Mean Local Gradient) MeLG method.

The two local gradient methods cannot be compared by their bounds, as they look at different metrics of the local gradients. Table 3 gives a sense of the number of time points identified by different bounds for each method. A bound of 4. 5 °C/m MeLG identifies a similar number of time points as a bound of 10 MxLG (2871 to 3176). Table 3 shows the persistence of thermal gradients on the structure. Out of the entire data set of 16,641 time points of measurement, there was only one time point where the maximum local gradient was under 7 °C/m, and there were no time points when the mean local gradient was under 3.5 °C/m. Figure 13 shows the same 3D signature as in Figure 4, with time points of mean local gradient under 4.5 °C/m in red.

The following conclusions can be carried out from the results shown in Table 4:

1. Each method above shows a better linear fit in terms of coefficient of determination and standard error than the total data set (without minimum gradient filters), for a number of points close to 1000 (i.e., 890) or more. This emphasizes the need of having a relatively large number of points to use, in which case there is an improvement in the accuracy of the best fit line.
2. The first component of the vector **n** is significantly different for MxLG with bound 8.0 °C/m (0.078) from any other MxLG bound shown in Table 4; this shows that there is an insufficient number of points used in calculating the vector **n** (only 112 points).
3. For all other MxLG methods, the first component of the vector is a stable value at 0.083–0.084, the second component is very stable at 0.988, and the third component is also stable at 0.132 ± 0.001. These values are very stable and provide confidence that any significant variation in any component of vector **n** from these values can be interpreted as unusual structural behavior in TD-SHM.
4. The value of the third component of vector **n** as determined from the entire data set is about 5% lower than if calculated using MxLG (0.126 vs. 0.132). Comparing the vector of MxLG with bound 9.5 °C/m to the total data set, a larger change in temperature (0.132 to 0.126) would result in a smaller change in strain (0.988 and 0.990) and the same change in displacement. This systematic difference in the third component of the vector of the best fit line shows that filtering out thermal gradients from the structure identifies a difference in the temperature–strain–displacement relationship.
5. The third component of the vector **n** is significantly different for MeLG with bound 4.00 °C/m (0.108) from every other MeLG bound shown in Table 2; this is to a lesser extent true for the first component too. These differences show that there is an insufficient number of points used in calculating the vector **n** using MeLG with bound 4.00 °C (380 points).
6. For MeLG with bounds 4.25 °C/m to 5.00 °C/m, the vector **n** of the BFL does not reach a consistent set of values. The first component has an upward trend from 0.077 to 0.080, increasing with each increase in bound, as does the third component. The vector begins to approach the vector of the total data set, showing that the larger bounds do not filter out thermal gradient effects.
7. For MeLG with bound 4.50 °C/m, the first component of the vector is approximately 8% lower from the same component from entire data set (0.078 vs. 0.084). The third component is also lower (for about 5%), but the second component is the exact same. This result is interesting, as the MeLG method requires a lower change in temperature to produce the same strain as from the normal vector. This difference highlights the difference in behavior identified by removing the influence of thermal gradients.

**Figure 14:**
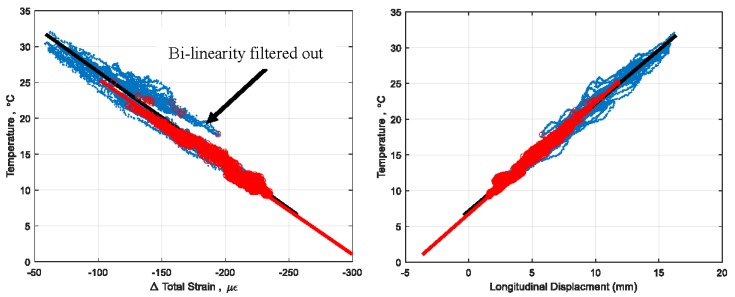
2D projections of 3D signature which significantly reduce the bi-linearity. Red indicates time points of minimal thermal gradient found using MeLG = 4.5 °C/m, and red line indicates best fit line for minimal gradient data set, as in Figure 13.

One strong result can be seen in Figure 14, where the MeLG metric filters out the bilinear behavior in the signature as the thermal gradients are filtered out. To emphasize this effect, 2D projections of the signature are given in Figure 14. The higher bounds of MxLG and MeLG show a slightly higher *R*^2^ than the total data set, possibly because as more points are identified the signature begins to stabilize, whereas smaller numbers of points can skew results. The two metrics have very similar goodness of fit statistics but vary more in the vector of the best fit line, describing different relationships between temperature, strain, and displacement. Both MeLG and MxLG show the capability to improve on the total data set. Future research will be able to show which method can be more useful for specific applications. 

### 4.3. MR and LG Comparison

**Figure 15:**
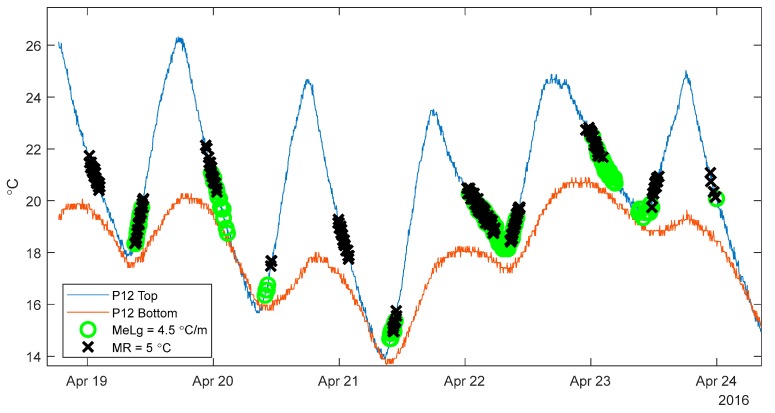
MR and MeLG Comparison, Pier 12.

MR and LG classes of methods filter time periods based on different metrics of minimizing thermal gradient, and identify different sets of time periods. To illustrate this, Figure 15 shows the time periods identified using both methods for five days in April 2016.

Figure 15 shows the time points identified as having a minimal thermal gradient over the entire structure, shown with two sensors for context. These are two of twenty sensors comprising the total data set on the structure. The bounds of each method in Figure 15 return a comparable number of points over the entire time period (2748 for MR = 4 °C and 2871 for MeLG = 4.5 °C/m) but identify different sets of time points. While this is not a strict rule, the MR tends to identify time points as the bridge is cooling down, while the MeLG tends to identify more time periods during heating. Figure 15 also shows some time periods where there is zero thermal gradient at Pier 12, but that are not identified as time periods of minimal gradients over the structure as other bridge sections remain with thermal gradients.

**Figure 16:**
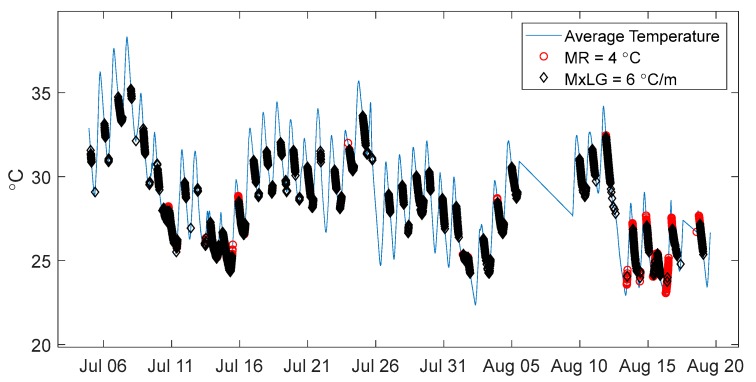
Summer 2010 Streicker Bridge minimal thermal gradient methods comparison.

Temperature data recorded on the Streicker Bridge in the summer of 2010 provides another example of the different sets of time points identified by the LG and MR methods. Due to incomplete data sets in the summer of 2016 (monitoring system disconnected for use in other projects), Figure 16 shows the summer of 2010 with time points of minimal thermal gradient, shown over the average temperature on the structure.

The MxLG identifies many more time periods in the summer than the MR method. This could be because of sunlight heating up certain parts of the bridge much more than others. The local gradients would not be overly affected as the top and bottom both heat up, but the overall temperature difference between different parts of the bridge longitudinally would exceed the maximum range. Table 5 shows a comparison of the MR and MxLG methods, by means of examining the number of time points identified during different seasons in 2010.

The MR identifies more time periods of uniform temperature in the fall than the MxLG. An MR of 3 °C identifies over a thousand less time points of minimal thermal gradient over the entire year compared to MxLG of 5 °C/m, but more time points in the fall. The MR identifies very few time points of minimal gradient in the summer, while the MxLG is fairly even in identifying time points over the entire year.

The differences in these methods can be seen when examining the relationships between the maximum range of temperatures on a structure and possible local gradients. For example, under an MR of 4 °C, the maximum possible local gradient would occur if that difference (4 °C) was present at one cross-section, creating a local gradient of 11.4 °C/m (using Equation (4) with an average h of 0.35 m). This level of thermal gradient would only occur if one cross-section was controlling the time period, with the maximum and minimum temperatures occurring at that cross section. A thermal gradient of 11.4 °C/m exceeds any bound set by the local gradient methods in Table 3. Conversely, using an MxLG of 9 °C/m would have a temperature difference of 3.15 °C at that cross section. While this value is lower than any MR bound used, the actual temperature values of different cross sections are not compared. The difference in the sets of data each method identifies as time points with minimum thermal gradients will provide distinct options for the basis of a model relating temperature change to strain and displacement, as can be seen in their different best fit line vectors.

The comparison shows that the two classes of methods are simply different rather than one being clearly superior. The two methods identify different subsets of the overall data, with the maximum local gradient method identifying many more times in the summer and maximum range in the fall, demonstrating the difference in the identification of minimal thermal gradients. The bounds chosen to be used within each method need to balance two competing necessities: the need for a large enough basis of time periods for the formulation of the temperature–strain–displacement model, and the need to minimize the thermal gradients in the identified time periods. The final choice of the method, or combination of methods, will depend on specific project application.

## 5. Conclusions

Temperature-Driven SHM considers temperature as the measurable input in SHM analysis. In order to clearly characterize the relationship between temperature as an input and strain and displacement as output, this paper proposes two classes of methods for identifying time periods of minimal thermal gradient across the structure. The Maximum Range (MR) method searches for instances where the range of temperature values on the structure is within some chosen bound. The Local Gradient (LG) methods compute the local vertical thermal gradient at each instrumented location in the structure, ensuring that each gradient is under some maximum gradient or that the mean absolute gradient is under the chosen bound. Each method employs different minimization criteria of thermal gradients in the structure, resulting in different subsets of time periods. These methods are validated quantitatively on a set of data taken from Streicker Bridge.

The MR methods showed a consistent improvement in the linear fit in terms of coefficient of determination and standard error of the 3D signature as well as a consistent vector of the line, different from the total data set, when there was a sufficient number of time points identified. Also, allowing a small amount of outlying temperatures (MR90, MR95) showed no degradation in the goodness of fit of the line, but allowed for an increase in the number of time points identified.

The LG methods showed an improvement on the linear fit of the 3D signature when identifying a substantial enough basis of time points (890 or over), and showed unique vectors of the BFL for each MxLG and MeLG. The LG methods also filtered out the bi-linearity shown in Figure 4, as the method removes time points of thermal gradient on the structure.

Each metric, the range of temperatures on the structure and the local gradients on the structure, found a linear temperature–strain–displacement relationship that is different from the total data set and has a stronger linear fit than the total data set. This vector of the line relating temperature, strain, and displacement, once thermal gradients are filtered out, will be sensitive to unusual structural behavior. The influence of errors in strain, temperature, and displacement monitoring to 3D signatures was considered out of scope and is not evaluated in this paper. However, detailed error analysis will be postulated and tested in the future work, when TD-SHM will be fully formulated and its performance assessed. One class of methods is not necessarily better in general than the other, and it will depend on the specific application which method is used. Future research on identifying time periods of minimal thermal gradient will be conducted in parallel with the development of TD-SHM.

## Figures and Tables

**Table 1:** Number of time periods identified by Maximum Range (MR).

Temperature Range (°C)	2.5	3	3.5	4	4.5	5
# Time Points in MR	0	15	438	2748	5967	8616
# Time Points 95% in MR	0	152	2773	6703	9779	11,341
# Time Points 90% in MR	4	1576	6989	10,104	11,519	12,228

**Table 2:** Best fit line statistics for MR method.

Bound	Metric	n	R^2^	σ	# Points
3.0 °C	MR	0.097 0.982 0.164	0.993	0.546	15
	MR95	0.079 0.989 0.123	0.994	0.810	152
	MR90	0.081 0.989 0.126	0.995	0.816	1576
3.5 °C	MR	0.079 0.989 0.128	0.993	1.003	438
	MR95	0.080 0.989 0.125	0.993	0.965	2773
4.0 °C	MR	0.081 0.988 0.130	0.994	0.968	2748
4.5 °C	MR	0.082 0.988 0.130	0.996	1.042	5967
**Total Set**		0.084 0.990 0.126	0.989	1.351	16641

**Table 3:** Number of time points identified by different methods using Local Gradient (LG) metric.

**Maximum Local Gradient (°C/m)**	7.00	7.50	8.00	8.50	9.00	9.50	10.00
**# Time Points under MxLG**	1	7	112	324	890	1919	3176
**Mean Local Gradient (°C/m)**	3.50	3.75	4.00	4.25	4.50	4.75	5.00
**# Time Points under MeLG**	0	26	380	1516	2871	4377	5844

**Table 4:** Best fit line statistics for LG methods, bounds in °C/m.

Metric	Bound	n	R^2^	σ	# Points
MxLG	8.00	0.078 0.990 0.125	0.983	0.564	112
	8.50	0.083 0.988 0.133	0.989	0.561	324
	9.00	0.083 0.988 0.132	0.992	0.643	890
	9.50	0.084 0.988 0.132	0.994	0.741	1919
	10.00	0.084 0.988 0.131	0.995	0.845	3176
MeLG	4.00	0.074 0.992 0.108	0.980	0.587	380
	4.25	0.077 0.990 0.119	0.992	0.584	1516
	4.50	0.078 0.990 0.121	0.994	0.636	2871
	4.75	0.079 0.989 0.123	0.994	0.750	4377
	5.00	0.080 0.989 0.126	0.994	0.904	5844
**Total Set**		0.084 0.990 0.126	0.989	1.351	16641

**Table 5:** Method comparison by number of points identified for different seasons, 2010.

# Time Points Identified, 2010					
MR			MxLG		
Bound (°C)	3.0	3.5	Bound (°C/m)	5.0	6.0
Year	1895	4582	Year	3002	6647
Summer	5	178	Summer	1143	2784
Fall	1875	4261	Fall	1788	3605
